# Comediation of Erythrocyte Haemolysis by Erythrocyte-Derived Microparticles and Complement during Malaria Infection

**DOI:** 10.1155/2020/1640480

**Published:** 2020-08-24

**Authors:** Ransford Kyeremeh, Samuel Antwi-Baffour, Max Annani-Akollor, Jonathan Kofi Adjei, Otchere Addai-Mensah, Margaret Frempong

**Affiliations:** ^1^Department of Medical Laboratory Sciences, School of Allied Health Sciences, College of Health Sciences, University of Ghana, P.O. Box KB 143, Korle-Bu, Accra, Ghana; ^2^Department of Molecular Medicine, School of Medical Sciences, Kwame Nkrumah University of Science and Technology, Kumasi, Ghana

## Abstract

**Background:**

Due to the sustained morbidity and mortality that malaria-associated anaemia imposes on patients, malaria is still a global threat, most especially, to residents in sub-Saharan Africa. Merozoite invasion and destruction of erythrocytes, a target for this study, have been necessary due to its unique nature and also since the erythrocytes suffer the most brunt of malarial infection leading to anaemia. The issue of malaria anaemia has to do with why uninfected RBCs get destroyed and even more so than infected ones. Studies have proposed that cytophilic anti-RSP2 (ring surface protein 2—merozoite rhoptry protein 2) antibodies present in sera enhance phagocytosis of RSP2-tagged RBCs by macrophages either directly or via complement, while others have proposed transfer of RSP2 to both infected and uninfected RBCs which may render them susceptible to phagocytosis. What is missing is the agent involved in the transfer of these parasite-induced surface proteins onto the uninfected RBCs, i.e., the mediator molecules. Considering the intracellular location of the parasite in the parasitophorous vacuolar membrane and the absence of a transport mechanism such as the Golgi apparatus within the mature RBC, since the latter has no nucleus, we propose that erythrocyte-derived microparticles (EMPs) may be the possible mediators.

**Aim:**

This study aimed at examining the immunological interactions between EMPs released during malarial infections and host erythrocytes that may lead to their lysis possibly through complement mediation.

**Methods:**

This was an experimental study during which malarial EMPs were isolated by differential centrifugation of malaria-positive plasma. This was followed by cell-based in vitro assays where malaria-positive EMPs were added to uninfected blood group “O” negative erythrocytes in the presence of complement and haemolysis checked for. *Results and Conclusion.* At a fixed volume of 50 *μ*L complement, there were statistically significant (*p* < 0.01) increases in mean percentage haemolysis as the volume of EMPs increased. Similarly, at a fixed volume of 50 *μ*L EMPs, there were statistically significant (*p* < 0.01) increases in mean percentage haemolysis with increasing volumes of complement. This was an indication that both complement and EMPs contribute significantly to uninfected erythrocyte haemolysis during malaria infection.

## 1. Introduction

Haemolysis refers to the abnormal breakdown of red blood cells (RBCs), which could be intravascular or extravascular [1]. There are numerous possible causes of haemolysis which may be due to defects within the red blood cells or abnormalities in the circulating environment as occur during parasitic invasion of the red blood cells [2]. Abnormal red blood cells are prematurely lost from the circulation in vivo because they are readily phagocytosed by macrophages [3]. This accelerated early break down of red blood cells invariably results in anaemia since it negatively affects the life span of the cells, causing their destruction before the normal physiological life span of 120 days [4]. Anaemia is thus defined as a reduction in haemoglobin concentration or circulating red blood cells below the normal reference ranges taking into consideration the age and sex of the individual and the altitude they find themselves [5].

During malaria infection, merozoite invasion and destruction of RBCs contribute significantly to ensuing anaemia where it is believed that the RBCs infected with malaria parasites beside haemolysing also release certain components that are transferred to other nonparasitized RBCs, leading to their phagocytosis and subsequent haemolysis as well. In fact, it is believed that, during malaria infection, the nonparasitized RBCs haemolyse more than the parasitized ones resulting in enhanced haemolysis leading to the ever present anaemia associated with malaria infection [6]. This leads to sustained morbidity and mortality making malaria to still be a global threat, most especially, to residents in sub-Saharan Africa [7].

Malaria is an acute and sometimes chronic vector-borne protozoan disease with widespread distribution in both tropical and subtropical regions [7]. The total burden of the disease as estimated by Ghana Health Service is over 3 million outpatient visits to public health facilities annually, with pregnant women and children forming the greater proportion [8]. The clinical manifestations of malaria anaemia emanate mainly from invasion and destruction of RBCs as well as host reaction to the malaria parasite infection. A variety of pathophysiologic mechanisms including increased deformation of parasitized and nonparasitized RBCs, increased splenic clearance, and haemolysis exacerbate anaemia [9]. Nonetheless, as stated earlier, the extent of RBC destruction, especially nonparasitized ones, is much greater than observed in other parasite-induced haemolysis. The basis of this is not completely known, though associated immune-mediated haemolysis has been postulated. Host-parasite interactions therefore offer opportunities for further research to clarify mechanisms that could be explored to fully understand why this happens for a better management of the disease.

Erythrocyte-derived microparticles (EMPs) have been found to be very important in malaria pathology, playing various roles in parasite-cellular interactions [10, 11]. Microparticles (MPs) are membrane-coated vesicles or submicron membrane elements of diameter of 0.1 to 1.0 *μ*m carrying molecules (when released) such as phosphatidylserine (PhtdSer) and tissue factors inherent in their parental cells [12–14]. They may also act as messenger molecules [15]. Proteomic analysis has also revealed that MPs from patients with systemic lupus erythematosus contain IgG, IgA, and IgM, an indication that the type of molecules carried by the MPs can be influenced by the disease condition involved [16, 17]. Again, the number of MPs can be highly increased in various pathological conditions such as sickle cell disease, antiphospholipid antibody syndrome as in systemic lupus erythematosus, multiple sclerosis, alpha-thalassaemia, and malaria [18].

Microparticles are derived constitutively from apoptosis and from activated blood cells [11]. They may carry, at their surfaces, molecules with proadhesive and procoagulant properties, suggesting that they could be implicated in the pathogenesis of the aforementioned diseases. Such a role has indeed been demonstrated in mice in which the involvement of procoagulant MPs in the development and growth of thrombi was assessed in vivo [19]. Also, in an experimental model of mouse malarial infection, a correlation between thrombocytopenia, increased number of platelet-derived MPs, and expression of cerebral complications whereby MPs promote sequestration of parasitized red blood cells (pRBCs) has been reported [19].

Mechanisms leading to haemolysis of infected RBCs include abnormal distribution of membrane phospholipids, such as phosphatidylserine, phosphatidylcholine, and phosphatidylethanolamine [20]. Phosphatidylserine is confined to the inner half of the membrane bilayer in normal RBCs, but it is exposed on the outer half in infected cells in conjunction with parasite maturation [20]. When PhtdSer is exposed on the outer half of the membrane bilayer, it can be recognized by macrophages as a signal for attachment and phagocytosis [21]. During acute *Plasmodium falciparum* malaria, RBCs can be detected that contain ring-infected erythrocyte surface antigen (RESA or Pf155) but no intracellular parasite [22]. This could represent an in vivo mechanism, whereby certain molecules from the infected erythrocytes are transferred to the noninfected cells through signal transduction [22]. This may cause many uninfected red cells to be destroyed in the spleen and possibly the liver, and their destruction has been identified as a major contributor to malarial anaemia [23].

In fact, both mathematical modeling and clinical observations suggest that 10 uninfected RBCs are removed from the circulation for each infected RBC that is removed [24]. The increased clearance of uninfected RBCs may be due to both extrinsic and intrinsic changes to the RBCs that enhance their recognition by phagocytes. These changes may include the deposition of specific molecules from the infected erythrocyte such as PhtdSer, immunoglobulin, and complement on the uninfected RBCs that may enhance receptor-mediated uptake by macrophages as well as complement-mediated lysis. Studies of the surface changes in RBCs of patients with severe malarial anaemia have shown that RBCs, whether infected or not, were more susceptible to phagocytosis [25–27]. This may be because of the parasite products deposited on the uninfected RBCs.

So, the question is how are these parasite products and other molecules deposit on the uninfected RBCs? Could it be that EMPs that are released from the infected RBCs transfer these complexes through signal transduction to the uninfected RBCs and hence render them susceptible to phagocytosis, haemolysis, and subsequently their removal? It has become prudent, therefore, for in-depth work to be carried out to examine candidate component EMPs to ascertain and understand their role in the pathogenesis of the disease. This work therefore looked at the immunological interactions between EMPs released during malarial infections and host RBCs that may lead to their lysis and thereby aggravating anaemia in malaria infection.

## 2. Materials and Methods

### 2.1. Study Design

The study was an experimental study conducted from January to October, 2018.

### 2.2. Study Area and Population

Blood samples were collected from the Central Laboratory of Korle-Bu Teaching hospital in the Greater Accra Region of Ghana, West Africa.

4 ml of human venous blood stored in EDTA tubes and tested to be malaria-positive at the Korle-Bu Central Laboratory was obtained from 10 outpatients who gave their informed consent.

### 2.3. EMP Isolation

Whole blood from each subject was first spun at a low speed of 160 ×g for 5 mins to separate RBCs from platelet-rich plasma. The platelet-rich plasma was in turn centrifuged at a higher speed (4000 ×g, 60 minutes) to obtain platelet-free plasma (PFP) and to remove any cell debris. The resultant supernatant was sonicated in a sonicating water bath (Townson and Mercer Ltd, Croydon) 5 times per 1 minute prior to further centrifugation in order to disperse aggregated exosomes. The supernatant was then centrifuged at 25,000 ×g for 90 minutes to pellet the EMPs. The EMPs were quantified and frozen.

The EMPs, now described as malaria-positive EMPs, were characterized and quantified (2.11 × 10^10^ EMPs/mL) using flow cytometry technique and stored at −80°C in 0.2 mL aliquots in Eppendorf tubes until they were used. Quantification allowed us to estimate the amount of EMPs in a volume of 50 *μ*L.

### 2.4. Complement-Rich Plasma Extraction from Guinea Pigs

Two guinea pigs were procured for the extraction of complement-rich plasma for use as a source of complement. Using a needle and syringe via a tail puncture, a total of 5 ml of blood was collected from the two animals into EDTA sample tubes and spun, and the complement-rich plasma was aliquoted into 0.2 ml Eppendorf tubes and frozen for use.

### 2.5. Preparation of Blood Group “O” Rh (D) Negative Red Cells for In Vitro Cell Analysis

A unit of donor blood group “O” Rh (D) negative whole blood (in order to avoid any serologic reaction) was obtained from the Korle-Bu Blood Centre after screening. Before use, the blood group was confirmed using the standard tube method of blood grouping. An aliquot was taken and washed four times in 0.85% phosphate-buffered saline and diluted to 3.0%.

### 2.6. Cell-Based In Vitro Analyses

Using 3.0% blood group “O” negative washed RBCs (prepared by diluting 0.3 ml of packed red blood cells with 9.7 ml of saline), an amount of malarial EMP suspension was added to uninfected (malaria-negative) blood group “O” negative RBCs at 37^o^C in the presence of complement and checked for haemolysis after 45 minutes. The experimental set up was done in duplicate, and the mean percentage haemolysis was determined for each set. Controls were set up using anti-C9 (commercially prepared anticomplement protein from Sigma-Aldrich).

## 3. Results

**Figure 1:**
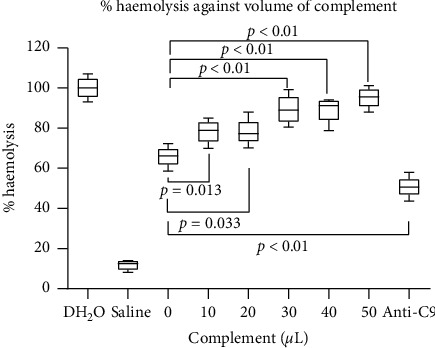
In vitro measurement of % haemolysis against EMP concentrations. From the figure, increasing EMP concentration results in increased haemolysis. This shows the extent to which variation in EMP concentration affected haemolysis when a fixed concentration of complement is used. Addition of anti-C9 however reduced percentage haemolysis drastically as shown, while using distilled water and saline (as controls), respectively, showed the highest and lowest levels of haemolysis.

At a fixed volume (50 *μ*L) of complement, there was an increase in mean percentage haemolysis when the volume of EMPs was increased. Using one-way ANOVA, the difference among them was statistically significant (*p* < 0.05). The post hoc analyses however showed no statistically significant differences in percentage haemolysis during the successive increases of EMP volumes from 10 *μ*L (*p*=1.00), 20 *μ*L (*p*=1.00), 30 *μ*L (*p*=1.00), and 40 *μ*L (*p*=1.00), though, at an EMP volume of 50 *μ*L, there was a statistically significant increase in mean percentage haemolysis (*p*=0.01). There was, however, a sharp drop in mean percentage haemolysis by about 50% when anti-C9 was added which was also statistically significant (*p*=0.01) (Figure 1).

**Figure 2:**
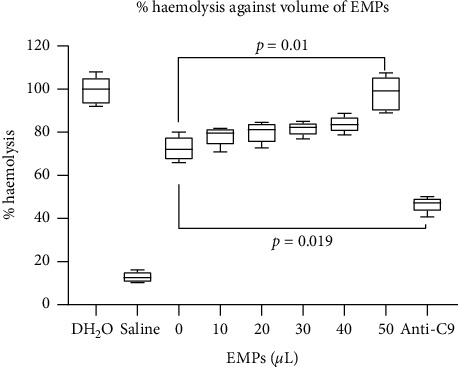
In vitro measurement of % haemolysis against complement concentration. From the graph, it can be seen that increasing complement concentration results in increased haemolysis. This shows the extent to which variation in complement concentration affected haemolysis when a fixed concentration of the EMP is used. Addition of anti-C9 however reduced percentage haemolysis drastically.

At a fixed volume of EMPs (50 *μ*L), there was increasing mean percentage haemolysis as complement volume increases. In multiple comparisons of the variation by one-way ANOVA, the difference among them was statistically significant (*p* < 0.05). The post hoc analyses also showed statistically significant differences in mean percentage haemolysis among the successive increases of complement volumes from 10 *μ*L (*p*=0.01), 20 *μ*L (*p*=0.03), 30 *μ*L (*p* < 0.01), 40 *μ*L (*p* < 0.01), and 50 *μ*L (*p* < 0.01) (Figure 2).

## 4. Discussion

Anaemia is a reduction in haemoglobin concentration or circulating erythrocytes below normal reference ranges taking into consideration the age, sex, altitude of the individual, and also the alteration of plasma volume, which can lead to false changes in haemoglobin concentration. Anaemia may result from either increased destruction or decreased production of erythrocytes. Malaria-associated anaemia, in particular, is a global health problem, causing enormous morbidity and mortality. Nonetheless, the extent of erythrocyte destruction, especially nonparasitized ones, is much greater than observed in other parasite-induced haemolysis. The basis of this is not completely known, though associated immune-mediated haemolysis has been postulated. Host-parasite interactions however offer opportunities for further research to clarify mechanisms that could be explored to fully understand why this happens for a better management of the disease. This study discusses how plasma membrane-derived extracellular vesicles and complement jointly induce erythrocyte haemolysis during malaria infection.

The increase in mean percentage haemolysis at a fixed volume (50 *μ*L) of complement while increasing EMP volume, as seen in Figure 1, shows that the latter plays a significant role in erythrocyte haemolysis during malaria infection. There was, however, a sharp drop in mean percentage haemolysis by about 50% when anti-C9 was added which was also statistically significant (*p*=0.01). This is an indication that though EMPs may directly or indirectly contribute to invasion and destruction/haemolysis of red blood cells during malaria infection, the associated mechanisms may involve complement activation as well. This is because during complement activation, the cytolytic process is greatly accelerated by the attachment of C9. That is, C5–8 polymerize C9 to form a tubule referred to as the membrane attack complex (MAC) traversing (destroying) the membrane. C9 is thus the ultimate orchestrator of complement-mediated cell destruction, and since addition of anti-C9 (a C9 inhibitor) subsequently reduced mean percentage haemolysis, the haemolytic event can also be said to be complement-mediated.

A similar event unfolded when a fixed volume of EMP (50 *μ*L) but increasing volume of complement was used when the erythrocytes were subjected to haemolysis (Figure 2). Addition of anti-C9 also reduced mean percentage haemolysis. Earlier study by Simona and colleagues showed that MPs mediate fibroblast growth factor-2 (FGF-2) release from cells [28]. FGF-2 is a polypeptide which regulates growth and differentiation of cells, but since it lacks a conventional secretory signal, its membrane release mechanism was not well established until MPs were found to play a role [28]. In another study involving the triggers of coagulation cascade, it has been shown that tissue factor-bearing microvesicles arise from lipid rafts and fuse with activated platelets to initiate coagulation [29]. Tissue factor (TF) is a type-1 transmembrane protein which serves as a cofactor for serine protease factor VIIa during the conversion of factor X to factor Xa which subsequently forms a complex with FVa in order to convert prothrombin to thrombin. This event, which occurs on the surface of anionic phospholipid provided by the activated platelet, is very significant in fibrin clot formation, the ultimate product of the coagulation cascade.

The present study proposes transfer of certain surface molecules/receptors by EMPs onto the surface membrane of uninfected red blood cells during malaria infection which render them susceptible to complement-mediated haemolysis. This is also in line with a study suggesting that platelet-derived microparticles are able to transfer various receptors including CXCR4 to CXCR4-null cells and render them susceptible to HIV infection [30], thus triggering a number of physiological responses, some of which are proliferation of cells, adhesion, and chemotaxis, as well as inducing metastasis and angiogenesis in the lung and breast cancer [31, 32].

## 5. Conclusion

From the findings of the study, it can be concluded that malarial MPs transferred certain surface molecules onto the surface membrane of the washed blood group “O” Rhesus (D) negative cells which facilitated their recognition by complement and hence resulted in their haemolysis. The drop in haemolysis after the addition of anti-C9 confirms the comediation of complement in haemolysis.

## Data Availability

The datasets used and/or analyzed during the current study are available from the corresponding author on reasonable request.
